# Modern Tools for Rapid Diagnostics of Antimicrobial Resistance

**DOI:** 10.3389/fcimb.2020.00308

**Published:** 2020-07-15

**Authors:** Antti Vasala, Vesa P. Hytönen, Olli H. Laitinen

**Affiliations:** ^1^Protein Dynamics, Faculty of Medicine and Health Technology, Tampere University, Tampere, Finland; ^2^Fimlab Laboratories, Tampere, Finland

**Keywords:** antibiotic resistance, antimicrobial susceptibility test, antimicrobial resistance, point of care test, rapid AST

## Abstract

Fast, robust, and affordable antimicrobial susceptibility testing (AST) is required, as roughly 50% of antibiotic treatments are started with wrong antibiotics and without a proper diagnosis of the pathogen. Validated growth-based AST according to EUCAST or CLSI (European Committee on Antimicrobial Susceptibility Testing, Clinical Laboratory Standards Institute) recommendations is currently suggested to guide the antimicrobial therapy. Any new AST should be validated against these standard methods. Many rapid diagnostic techniques can already provide pathogen identification. Some of them can additionally detect the presence of resistance genes or resistance proteins, but usually isolated pure cultures are needed for AST. We discuss the value of the technologies applying nucleic acid amplification, whole genome sequencing, and hybridization as well as immunodiagnostic and mass spectrometry-based methods and biosensor-based AST. Additionally, we evaluate the potential of integrated systems applying microfluidics to integrate cultivation, lysis, purification, and signal reading steps. We discuss technologies and commercial products with potential for Point-of-Care Testing (POCT) and their capability to analyze polymicrobial samples without pre-purification steps. The purpose of this critical review is to present the needs and drivers for AST development, to show the benefits and limitations of AST methods, to introduce promising new POCT-compatible technologies, and to discuss AST technologies that are likely to thrive in the future.

## Background and Foreword

There is an unmet need for rapid and decentralized diagnostics in outpatient clinics to reduce the misuse of antibiotics. It is important to identify the etiological pathogen and to differentiate between viral and bacterial infections, to identify the antimicrobial resistances in microbes, and to find out which antimicrobial agent should be used for the cure. Thereby the unnecessary use of antibiotics could be minimized and the spread of antibiotic resistance better controlled. According to the WHO, antimicrobial resistance (AMR) is the largest global health threat in the 21st century and requires urgent measures. Common infections are becoming untreatable due to the emergence of AMR. More than 700,000 people die of drug-resistant infections every year, and this figure is expected to reach ten million by 2050 (United nations meeting on antimicrobial resistance, 2016). According to current understanding, in EU and EEA countries more than 33 000 people are killed every year due to antibiotic-resistant bacteria. They also cause close to 900 000 disability-adjusted years (Cassini et al., 2019). Bringing diagnostics closer to the general practitioners and hence to the patient would cause a paradigm shift from empirical to evidence-based treatments of infectious diseases in outpatient clinics. Rapid diagnostics are needed for both pathogen identification and resistance testing. The prevalence of AMR may be very high for some species in certain geographic locations. According to the current recommendations on AST (antimicrobial susceptibility testing), pure culture isolates are used to test the effect of antimicrobial drugs. This is required as the sample matrix (blood, urine, mucosal) as well as the number and proportions of different microbial species may vary a lot in polymicrobial samples. It may be unclear whether the detected microbe is pathogenic or merely a commensal species. Despite significant progress in diagnostic technologies in recent years, most patients with infectious diseases are still treated empirically and thus antibiotics are heavily overused (Li et al., 2016; Mashalla et al., 2017). Even in Western countries, 30% of antibiotic prescriptions are considered to be either unnecessary or suboptimal (Centers for Disease Control Prevention, 2018). Current diagnostic tests serve hospitalized patients rather well, but they are often not available in outpatient clinics. For typical growth-based AST, several cultivation rounds are required: enrichment cultivations (e.g., blood cultures) to increase the number of bacteria, plate cultivations to obtain pure cultures, and finally AST for liquid or plate cultures using various antimicrobial loads. Microbiology laboratories apply the EUCAST-accepted breakpoint values to define whether the microbe is susceptible or resistant to the tested antibiotic. They use the disk diffusion method or other systems calibrated to EUCAST standards. Altogether, AST may require several days. Rapid molecular diagnostics has been discussed in many excellent reviews (Pulido et al., 2013; Plüddemann et al., 2015; Li et al., 2017b; Maurer et al., 2017; Syal et al., 2017; Maugeri et al., 2019). They present the progress in Nucleic Acid Amplification Technology (NAAT), electrochemical methods, microarrays, micro- and nanoparticles, as well as mass spectrometry applications, but also emphasize that very few of the molecular methods have acquired FDA approval.

The review of David Boyle, “Tuberculosis Diagnostics Technology Landscape” is worth reading, since, although not focusing on AST, it presents comprehensively new affordable molecular diagnostic technologies available in standard microscopy stations, particularly in developing countries (Boyle, 2017). The most up-to-date and concise progress compendia in the field of AST can be found in congress presentations, lectures and webinars. Prof. Mark Fisher's webinar “Rapid Antimicrobial Susceptibility Testing” (ARUP, 2020; Scientific Resource for Research and Education: Educational Resources—Rapid Antimicrobial Susceptibility Testing | University of Utah) is particularly useful.

The innovations in electronics, biosensor techniques, optics, microfluidics, hybridization technologies and DNA amplification technologies have yielded new approaches in AST. Unfortunately, the scientific papers on these technologies do not sufficiently relate these findings to the practical needs in POCT. The requirement of a microbiology laboratory and the time and resources needed for the enrichment of cultures, preparation of pure cultures. and sample treatments is often not sufficiently considered. These requirements also easily blur the total costs of AST. For these reasons, the deployment of new molecular methods for AST has been very slow (Doern, 2018). The standards of care for antibiotic prescription are quite consistent in most European countries and the USA and apply evidence-based ID and AST when available. In total, urinary and respiratory tract infections form a significant part of acute infections. Quick and accurate diagnosis for these diseases already at outpatient settings could efficiently restrict the spread of AMR bacteria and allow an early isolation of the carrier and correct treatment. Rapid diagnosis would also allow the prompt dismantling of unnecessary patient isolation, saving money and resources. However, technical improvements translate into benefits only if the structured communication and interpretation of the results are applied by the clinicians (Maurer et al., 2017) and the cost of these technologies are reasonable.

Vital emergency diagnostics for septicaemia has received a lot of resources (Marco, 2017; Hughes, 2018). New sensitive methods such as T2MR (T2Biosystems, USA) can quickly detect molecular targets directly from clinical samples, enabling rapid pathogen identification and detection of resistance factors. However, growth-based AST for blood requires a fairly high bacterial count for enrichment cultures and a well-equipped microbiology laboratory. The achievements in blood testing do not necessarily relate well to the antimicrobial stewardship in the front-line: healthcare settings.

Optimal antimicrobial therapy policy would require (1) Fast point of care analysis, (2) Identification of the etiological agent, (3) Finding of an efficient antibiotic, and (4) Determination of the functional dosage. According to Prof. Gunnar Kahlmeter (Chairman, EUCAST general committee) (Kahlmeter, 2016), the key questions for any new AST technology are:

- Is it generally applicable or suitable only for one infection (for example sepsis or one resistance type)?
- What is the capacity? How many organisms/agents per hour can be processed?
- Has the technology been validated against reference methods?
- Are there any reference installations?
- Is scientific literature available?
- When will it be on the market?

Complete answers are hard to dig up, but this review tries to address these questions. We present the needs and drivers for AST development, increase the understanding about the role of rapid AST in diagnostics of infectious diseases, show the benefits and limitations of AST methods, introduce the key POCT-compatible technologies, and contemplate on which AST technologies are likely to thrive in the future.

## Current Technologies in Antimicrobial Susceptibility Testing And Microbial Identification

Bacteria can acquire resistance to antibiotics by several mechanisms. The antibiotic can be degraded or chemically modified (by acetylation, phosphorylation, nucleotidylation, ADP-ribosylation, mono-oxygenation, glycosylation). The drug intake can be prevented, or efflux can be enhanced. Some resistance mechanisms are based on the reprogramming of cell wall synthesis. Even slight changes in the target molecule, e.g., a point mutation in the ribosomal protein, can render the antibiotic inefficient. The overwhelming variety of antimicrobials and resistance mechanisms complicates AST. Genotypic (nucleic acid-based) methods can only find resistances that are searched for, and the potentially found resistance genes are not necessarily from the actual pathogenic organism. According to EUCAST and CLSI guidelines, reliable antibiotic resistance diagnostics requires phenotypic testing, i.e., an experimental test whether the microorganism grows in the presence of the antibiotic. These methods work regardless of the resistance mechanism and give answers to the practical questions: which antibiotic is efficient and which dose should be applied in the therapy. Classical AST techniques such as broth microdilution, disk diffusion, gradient tests, agar dilution and breakpoint tests are based on continuous exposure of a bacterial isolate to a set of antimicrobials, followed by a visual detection of growth. The use of advanced optoelectronic systems, fiber optics, microfluidics and indicator dyes sensitive to redox-state or pH can further enhance the sensitivity and performance of optical systems.

Several commercial systems have streamlined and partly automatized the follow-up of AST cultures. Systems like Vitek and Microscan perform automated turbidity measurement for multiwell liquid cultures. BD Phoenix system™ applies a redox indicator to enhance the detection of organism growth. These systems have turnaround times as short as 4 h for ID and 6–8 h for susceptibility testing (She and Bender, 2019). The CE-marked Alfed 60 AST™ system (Alifax, Italy) uses sensitive laser-light scattering technology to detect bacterial growth in a liquid culture broth and provides antimicrobial susceptibility results directly from positive blood culture bottles within 4–6 h.

Such broth dilution-based systems use ready-made AST cassettes or cards containing positive controls and wells with increasing concentrations of antibiotics. They provide continuous growth monitoring and can analyze MIC patterns for a large group of organisms through their extensive databases.

Pathogen identification (ID) is usually a preliminary step of AST. For blood samples, microscopy and Gram-staining are nearly always performed, as Gram-positive bacteria in general have a more limited variety of antibiotic resistances and less problems with multidrug resistance. In AST, the following sequence is typically applied: First clinical samples are cultured to obtain pure isolates. Then identification (with MALDI-TOF mass spectrometer, if available) is performed. Thereafter, AST and MIC determination is performed according to EUCAST or CLSI standards. This sequence, in total, requires several days. Standard healthcare settings do not have advanced microbiology laboratories with mass spectrometry instruments. Their arsenal for the diagnosis of infectious diseases may be limited to immuno-chromatographic strip tests (aka lateral flow tests = LF or “dip-sticks”) applied to the detection of viruses (e.g., influenza) and bacterial pathogens causing sexually transmitted diseases.

Quick identification can efficiently restrict the search palette for certain antibiotics. Hence mass spectrometry has become a versatile workhorse in clinical laboratories. It is routinely applied for bacterial ID as soon as isolated colonies are available. Through the simultaneous measurement of several metabolites a biochemical signature of microbes can be obtained. Matrix-assisted laser desorption/ionization time of flight (MALDI-TOF) applies laser energy to evaporate the matrix-bound sample, that is then immediately analyzed. When frequent sampling is applied, MALDI-TOF can even provide semi-quantitative growth rate data (Maxson et al., 2017). Bruker Corp. (Germany) has launched test kits such as BT STAR-Carba Assay for AST based on antibiotic degradation monitoring.

AST for blood cultures has been applied after a short-term cultivation on agar plates followed by susceptibility testing using VITEK AST cards selected on the basis of MALDI-TOF analysis (Idelevich et al., 2014; Mauri et al., 2017). By applying the MBT-ASTRA™ test with MALDI Biotyper for ID and AST, identification of mycobacterial strains resistant to rifampicin, isoniazid, linezolid, ethambutol, clarithromycin and rifabutin can be obtained 1 week faster than through routine cultivation-based AST (Ceyssens et al., 2017). The MS approaches combined with NAAT or microfluidics will be presented in “Future technologies” section.

## Current Technologies for Rapid AST

**Figure 1:**
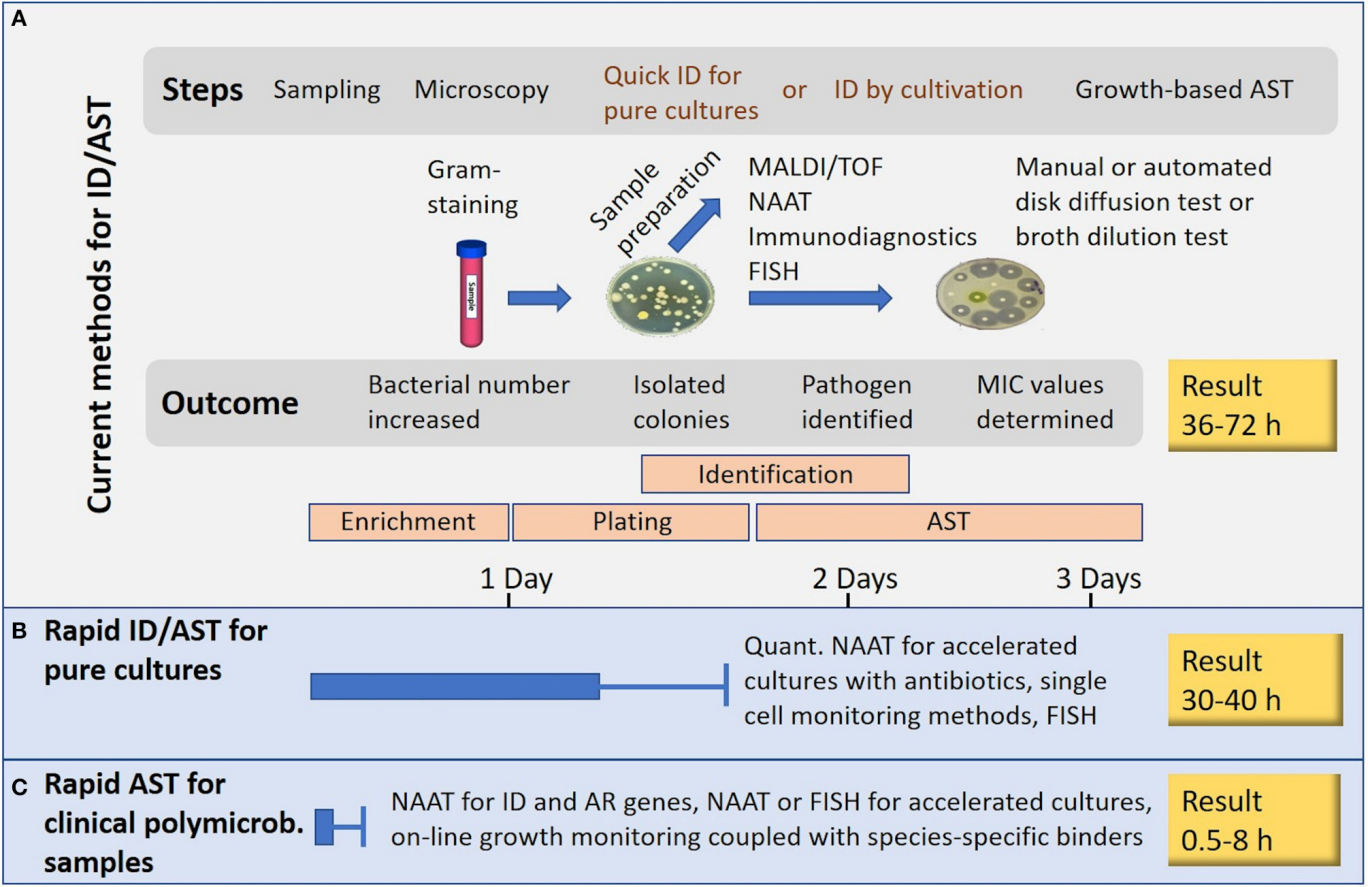
Rapid AST or rapid result? **(A)** Current technologies. **(B)** Rapid AST applicable to pure cultures. **(C)** Rapid AST for clinical polymicrobial samples. The presented times are rough estimates and generalizations.

**Figure 2:**
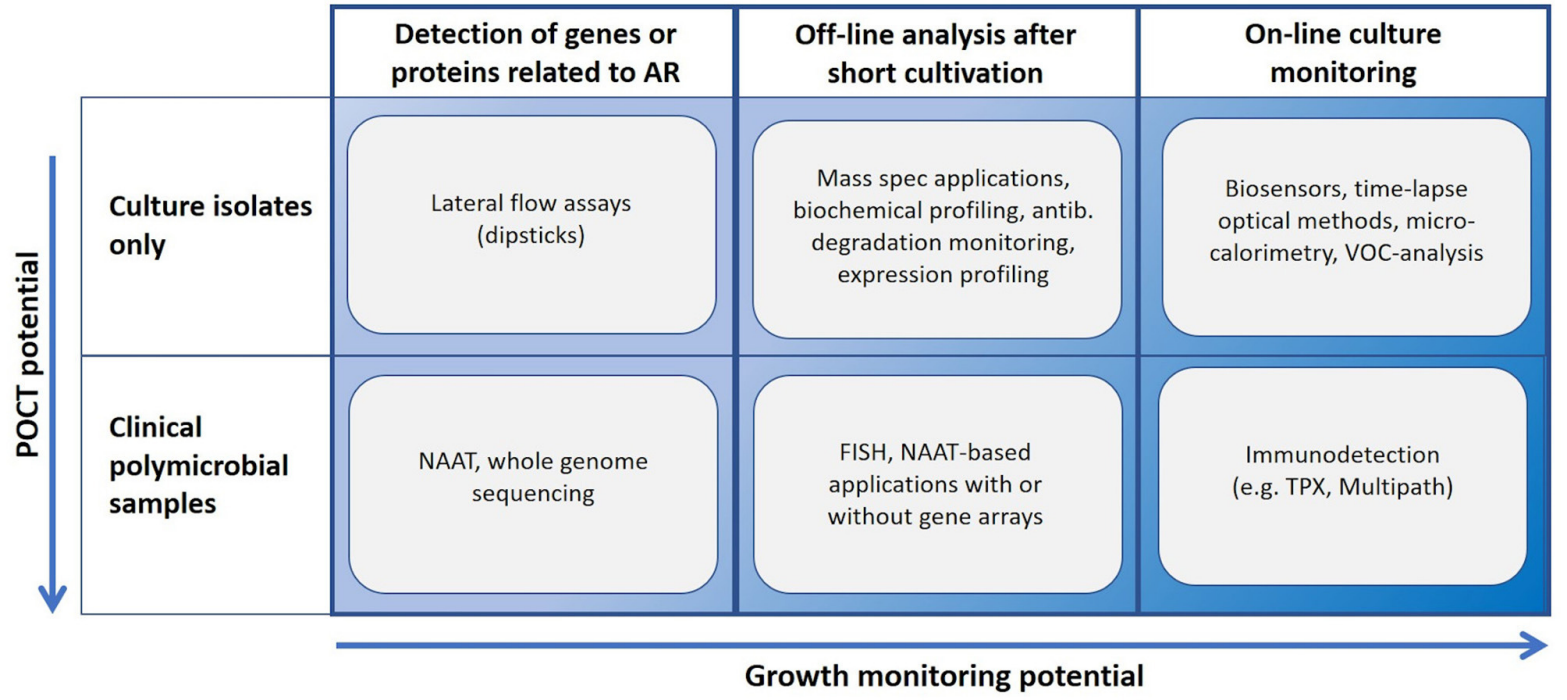
Usability landscape of rapid AST technologies. NAAT, nucleic acid amplification technology; TPX, immunodetection based on two-photon excitation fluorometry; Multipath, immunodiagnostic method applying magnetic spheres for cell separation, fluorescent nanoparticles for labeling and non-microscopic imaging.

Many novel methods claim to perform AST in minutes or in few hours. Such statements usually ignore the need of time-consuming steps such as enrichment cultures and isolation of pure cultures (Figure 1). Methods based on NAAT, nucleic acid hybridization or immunodiagnostics in principle allow the use of non-purified polymicrobial clinical samples. A short cultivation with a pre-determined antibiotic load followed by NAAT (e.g., isothermal amplification) can reveal AR, and even provide a rough estimate of the minimal inhibitory concentration (MIC) for the tested antibiotics. Most rapid growth-based AST methods perform end-point analysis only, whilst others rely on frequent sampling from the cultivation chamber. Some sensitive immunodiagnostic systems however provide real on-line growth monitoring (Figure 2). Biosensor technologies detecting changes in microbial metabolism, movement or heat production have not yet provided convincing clinical demonstrations. Fast, reliable, easy-to-use and inexpensive systems applicable to AST in outpatient clinics are still elusive (van Belkum et al., 2019a).

### Microscopy

Counting of bacteria on agar plates by microscopy techniques is possible long before they have reached the number providing visible colonies. *E. coli* colonies visible by eye contain roughly 5 × 10^6^ bacteria, but by microscopy microcolonies formed by 120 cells can already be detected (London et al., 2010). Drug susceptibility (MODS) for *Mycobacterium tuberculosis* can be assessed by observing cell aggregates (cords) microscopically in sealed microtiter plates (den Hertog et al., 2014). The Growth Direct System by Rapid Micro Biosystems Inc. detects microcolonies with digital imaging by illuminating them with blue light and directing the cellular autofluorescence directly onto a CCD chip without magnification. The average time for *E. coli* detection by this autofluorescence was 3.1 h compared to an average of 8.5 h for the visual plate counting method. Although the idea of applying this to AST has been declared in the patent application, clinical studies for AST have not yet been presented.

Automated microscopy systems can provide real time growth curves, and quantitative bacterial counts have been presented. Multiplexed automated digital microscopy (MADM) applying Fluorescent *in situ* Hybridization (FISH) has been commercialized by Accelerated Diagnostics (USA) for rapid on-line AST (Metzger et al., 2014; Chantell, 2015). The Accelerate Pheno® system can separate impurities from clinical samples (e.g., blood or urine) by brief electrophoresis, which runs impurities into a gel. After this a change of the electric field polarity repels the microbes back to the liquid. A fluorescence signal is detected every 10 min from samples taken from the bacterial culture multiplying in Mueller-Hinton media (Charnot-Katsikas et al., 2017). This system seems to be currently the only FDA-approved growth-based rapid diagnostic AST system (Doern, 2018). The performance of Accelerate Pheno® has been demonstrated with many clinical studies, e.g., with urinary tract infections (Charnot-Katsikas et al., 2017) and bloodstream infections (Charnot-Katsikas et al., 2017; Marschal et al., 2017; Descours et al., 2018). With 232 positive blood cultures tested, the overall essential agreement with routine methods was 95.1%, and the time needed for AST was decreased by 42 h in comparison to standard growth-based analysis. ID could be obtained in 1.5 h and AST in 7 h (Charnot-Katsikas et al., 2017).

### Hybridization-Based Systems

FISH is a highly specific method to visualize the presence of the target organism in a quantitative manner. The PNA-FISH technology applies peptide nucleic acid probes which allow more rapid and specific binding than DNA or RNA probes (Perry-O'Keefe et al., 2001; Almeida et al., 2009; Cerqueira et al., 2011). It is applied in the commercial QuickFish technology (OpGen, USA) which performs ID by targeting 16S rRNA (Enroth et al., 2019). XpressFish specifically detects the *mecA* gene in *Staphylococcus*, allowing, when used subsequent to QuickFish-based identification, diagnosis of methicillin resistance already 2 h after the blood culture turns positive (Salimnia et al., 2014). Since a temperature of 55°C is needed for target cell permeabilization, fixing and hybridization, PNA-FISH systems are not applicable to on-line growth monitoring. FDA-approved systems are available also from bioMérieux (Durham, NC). Clinical laboratories applying mass spectrometry are unlikely to need PNA-FISH technology; the Bruker MALDI Septityper® kit PBP2A, e.g., can detect the *mecA*-encoded PBP2A-protein in 1 h with fairly low costs.

In actively growing cells, RNA is more abundant than DNA and thus a good target for probing. Especially precursor rRNA (pre-rRNA), the intermediate stage in the formation of mature rRNA, is a good indicator of bacterial metabolism, viability, and growth rate (Halford et al., 2013). The biosensor-based AST (b-AST) system from Genefluidics Inc. (CA, USA) measures bacterial growth by quantifying 16s rRNA molecules with an electrochemical biosensor. This system uses species-specific probes and integrates nanotechnology, plastic micro electromechanical system and microfluidics (Mach et al., 2011). This system achieved a detection limit of 10^4^ cfu ml^−1^ in rapid AST of clinical urine and blood samples (Liu et al., 2014). Mohan et al. demonstrated the simultaneous detection of uropathogens and the host biomarker lactoferrin in urinary tract infection, but reached only 89% sensitivity in the pathogen identification (Mohan et al., 2011). In 2018 Genefluidics announced CE-IVD Marking for the UtiMax™ kit, which provides ID in 30 min and AST in 2 h from urine with an overall sensitivity of 100% and specificity of 98.2% (GeneFludics Inc., n.d.).

### Nucleic Acid Amplification Technology (NAAT) in AST

The first generation “molecular tests” such as restriction fragment length polymorphism, pulsed-field electrophoresis, multiple locus tandem repeat analysis, multi-locus sequence typing and virulence genotyping were suitable rather for typing and outbreak investigation than for AST. Since these technologies require a high amount of purified nucleic acid, they do not allow rapid diagnostics. However, hybridization-based approaches and molecular beacon systems have persisted and are creatively combined with NAAT technologies.

NAAT is a very powerful tool for pathogen identification, especially when combined with a syndromic approach. Many diagnostic panels provided e.g., by BioMérieux, Elitech, Bosch, Eplex, Qiagen, or Becton Dickinson, include detection of specific AR-genes. They can provide clinically relevant results, especially in cases where a detailed antibiogram is not needed. For example, pathogen such as *Bordetella, Legionella, Mycoplasma, Chlamydia trachomatis*, or *Neisseria gonorrhea* exhibit quite few antibiotic resistances. The detection of specific AR genes, however, cannot give an undisputed proof of antibiotic resistance. The identified AR genes do not necessarily relate to the pathogen causing the disease, or the found resistance gene may not be functional. NAAT neither defines the MICs nor directly indicates which antibiotics should be used. An advantage of NAAT is that the tests can be relatively quickly updated for newly emerging pathogens and resistance factors. Quantitative PCR (qPCR) allows a rough quantification of microbes. Quantitative reverse transcription PCR (qRT-PCR) can additionally assess the expression level of resistance genes after exposure to different antibiotic loads and thus provide rough MIC values. The cost of devices and reagents for qRT-PCR are unfortunately currently far beyond the level acceptable for routine AST. NAAT is a powerful tool for the identification of both bacterial and viral pathogens. In principle it allows the use of patient samples without enrichment cultivations. However, due to the risk of losing the template during nucleic acid extraction and the sensitivity of DNA polymerases to impurities in the sample matrix, enrichment cultivations and nucleic acid purification are often necessary. Due to the vast choice of different fluorescent labels, several target genes can be conveniently tested in parallel from the same sample. The systems can reach further sensitivity and specificity by applying hybridization to DNA arrays. A good number of FDA-approved multiplexed diagnostic panels are already available. Such commercial systems include Xpert® (Cepheid Inc.), ePlex (GenMark Diagnostics), Unyvero (Curetis AG), BD Max (Becton-Dickinson), SeptiFast (Roche), Magicplex (SeeGene), Novodiag (Mobidiag), and GenomEra (Abacus Diagnostics). The Xpert® technology combines sample preparation, real-time PCR and nucleic acid analysis with molecular beacons. The ePlex system provides electrochemical detection of the amplified sequences, wherein the detection is achieved by hybridizing ferrocene-labeled probes with the sample DNA (Nijhuis et al., 2017). Hybridization techniques and microfluidics have been implemented also to Novodiag's GenomEra microarray platform. It applies time-resolved fluorescence detection of the amplified product on a sealable plastic chip packed with dry chemistry. Also Curetis, Becton-Dickinson, Roche and SeeGene products apply cartridge-based integrated designs (Hughes, 2018). Fast analytics of PCR products enhance the throughput of NAAT systems. T2Biosystems has recently launched a test panel able to detect 13 resistance genes from both gram-positive and gram-negative pathogens directly from blood. The amplification products are detected by magnetic resonance after hybridization with DNA probes conjugated with superparamagnetic particles (Hong Nguyen et al., 2019).

PCR/electrospray ionization–mass spectrometry (IRIDICA PCR/ESI-MS by Abbott Laboratories Inc., USA) allows the detection of >750 different bacterial species in a single test (Strålin et al., 2016). The High Resolution Melting system (HRM, by ThermoFisher) identifies variations in nucleic acid sequences by detecting small differences in PCR melting curves. A melting curve analysis for real-time quantitative PCR or digital PCR (wherein the sample is partitioned into a large number of individual wells each containing either 1 or 0 targets) performed for growing bacterial cultures, revealed both ID and antimicrobial susceptibility profiles for *E. coli, E. faecalis, P. mirabilis* and *S. aureus* in ~6.5 h, when analyzed by machine learning algorithms (Athamanolap et al., 2017). For routine analytics, such systems are still too expensive, and they require separate kits for DNA extraction and PCR. However, new enzymes and technologies such as ligase chain reaction (LCR) (Barany, 1991), nucleic acid sequence-based amplification (NASBA) (Compton, 1991), strand displacement amplification (SDA) (Walker et al., 1992) and loop-mediated isothermal amplification (LAMP) (Notomi et al., 2000) have simplified nucleic acid amplification, made it more robust (often allowing the use of samples without NA extraction), enabled miniaturization and cut down the costs of instrumentation by allowing NAAT at a constant (isothermal) temperature. NUCLISENS® EASYQ® (bioMerieux) was the first automated system to combine NASBA and real-time detection using molecular beacon probes. It enabled the fast detection of *Klebsiella* carbapenemase genes (Spanu et al., 2012). LCR has been successfully used for the detection of ciprofloxacin and doxycycline resistance genes in *Bacillus anthracis, Francisella tularensis*, and *Yersinia pestis* (Oblath et al., 2013). LCR can be easily integrated into detection systems such as electrochemical and magnetic biosensors, quantum dots, quartz crystal and leaky surface acoustic surface biosensors, Surface Enhanced Raman Scattering (SERS), chemiluminescence and fluorescence resonance energy transfer (Oblath et al., 2013). High-throughput multiplex genotyping can be achieved also by DNAzyme (DNA oligonucleotides) technology, rolling circle amplification (RCA) and strand displacement amplification (SDA) techniques. LAMP is especially robust, since it is less sensitive to inhibitors than standard PCR. This allows analysis after a minimal processing of blood (Curtis et al., 2008), urine or stool (Francois et al., 2011). LAMP is applicable to low-resource field settings where DNA or RNA extraction is not possible. However multiplexing approaches are less developed for LAMP than for PCR (Sahoo et al., 2016).

### Immunodetection of Pathogens

Immunodetection is a specific and sensitive method for the identification of bacterial pathogens (Verma et al., 2013), toxin proteins (Zhu et al., 2014), and viruses. Since immunodetection does not necessarily require disruption of the target microbes, it can potentially provide pathogen identification and growth monitoring in a single step. It is applicable as simple lateral flow (LF) tests, but can also be integrated to biosensor technology, microfluidics and even to DNA/RNA-based analysis. Companies like Mizuho Medy, Alere, and Beckton Dickinson have launched several easy-to-use stick tests for clinical diagnostics of (influenza) viruses and bacteria causing sexually transmitted diseases. The binders are typically antibodies that are immobilized onto strips, micro/nanoparticle beads or biosensor surfaces providing an efficient and specific target binding. The detection antibody can be labeled with fluorescent dyes or redox enzymes to provide a quantitative signal.

Only few products are available for the direct detection of antibiotic resistance proteins. The LF-test developed by Kitao et al. detects chloramphenicol resistance in *P. aeruginosa* samples (Kitao et al., 2010). Alere Inc. has launched an immunochromatography test for the detection of MRSA, based on a PBP2a-specific chicken IgY antibody (Yamada et al., 2013). The PBP2a SA Culture Colony Test can identify MRSA in 6 min (Trienski et al., 2013; Delport et al., 2016). Coris Bioconcept (Belgium) has launched tests for the detection of carbapenemases (OXA-48-like, KPC, and NDM type) from enterobacterial isolates (Bogaerts et al., 2013; Glupczynski et al., 2017). Boutal et al. have presented LF tests for the carbapenemases TX-M-15, NDM, OXA-48-like, KPC, IMP, and VIM (Boutal et al., 2017). LF tests work well with isolated clinical isolates. They are, however, generally not applicable for direct analysis of clinical samples. The commercial MultiPath™ platform (First Light Diagnostics Inc., USA) applies non-magnified digital imaging for the detection of biomolecules tagged with antibody-coated fluorescent nanoparticles. The mariPOC® system by ArcDia Ltd, based on Two-Photon eXcitation fluoroscopy technology (TPX), is already in diagnostic use for immunogenic detection of pathogens. These two technologies will be discussed closer in the following sections.

## Future Technologies Under Commercialization

Mass spectrometry is likely to become tightly integrated into other AST technologies, especially in the diagnostics of septicaemia. In the MALDI-TOF Direct-On-Target Microdroplet Growth Assay (DOT-MGA), sample droplets (culture plus antibiotics in 6 μL volume) are spotted directly onto disposable MS-target plates, incubated for 3–4 h and then analyzed with MS (Idelevich et al., 2018). Screening panels for ESBL and AmpC β-lactamases of enterobacteria are already available (Correa-Martínez et al., 2019). The fast progress in microfluidics, biosensor technologies, isothermal amplification-based NAAT, and immunodetection has recently provided several potent systems which may eventually change the paradigms of AST.

Gradientech's QuickMIC system combines microfluidics with automated time-lapse photomicrography to follow growth inhibition along a linear drug gradient. It measures the greyscale intensity changes in the images caused by the formation of microcolonies and provides AST in 2–5 h (Malmberg et al., 2016). The system is currently seeking CE-IVD marking and FDA approval for AST in blood samples.

Q-Linea ASTar® applies time-lapse microscopy to fully automated monitoring of blood cultures as well as preparation and monitoring of bacterial isolates. It processes 12 samples at a time and 50 samples a day, delivering true MIC values for up to 48 antibiotics within 6 h. The system does not perform ID, but it can be connected to any ID system. Clinical trials will start in 2020.

The BacterioScan 216Dx system (St. Louis, MO, USA) measures both a sample's optical density (OD) and the scattered intensity by forward laser light scattering, allowing 10–100-fold higher sensitivity compared to normal OD measurements. This system can process 16 samples simultaneously and perform real-time continuous growth measurement. It can detect bacterial growth in 3 h from clinical urine samples containing >10^4^ cfu/ml of *E. coli, Staphylococcus aureus, Pseudomonas aeruginosa, Bacillus antracis, Yersinia pestis*, or *Burkholderia pseudomallei* (Bugrysheva et al., 2016; Hayden et al., 2016; Montgomery et al., 2017). The BacterioScan 216R Rapid AST System is currently under testing.

The oCelloscope™ system (BioSense Solutions, Denmark) applies digital time-lapse angled field microscopy and image analysis for standard 96-well plates. The time-to-result for positive blood cultures ranged from 1 to 4.2 h (Fredborg et al., 2015). However, the overall performance for AST has not yet been sufficiently tested.

Changes in individual cell morphology or size indicate growth long before bacteria have multiplied. Single-cell morphological analysis (SCMA) uses bright-field microscopy to determine antibiotic-induced changes in cells immobilized on an agarose channel chip (Choi et al., 2014). The commercial MultiPath™ platform applies non-magnified digital imaging for the detection of biomolecules tagged with antibody-coated fluorescent nanoparticles. Antibody-coated magnetic particles are used to bind to target cells and to pull them down to the camera surface, thus eliminating background signal and enabling a wash-free assay for clinical samples. The system counts individual targets in large areas and performs growth monitoring for multiple targets cells and enables the determination of MIC values. The MultiPath™ system is currently seeking FDA clearance.

The Two-Photon eXcitation fluoroscopy technology (TPX), commercialized by ArcDia Ltd. (Finland), allows separation-free detection of biological molecules in small reaction volumes by immunodetection (Vakkila et al., 2015). The mariPOC® test system was developed for rapid pathogen identification. It applies polystyrene microparticles as solid carriers for immunocomplex formation, that leads to three-component immunocomplexes (monoclonal antibody—antigen—labeled monoclonal antibody) on the microspheres in proportion to the analyte concentration. The detection of the immunoassay fluorescence signal is achieved by two-photon excitation from the surface of individual microspheres. This mechanism allows the use of unpurified clinical samples and on-line monitoring of viable cells in AST cultures. At present this system is under clinical testing for AST.

Nanostring Technologies Inc. combine genotypic and phenotypic AST through RNA detection. The GoPhAST-R platform detects mRNA expression signatures in bacteria after antibiotic exposure. The system can be used directly for positive blood culture bottles. It couples machine learning analysis of transcriptional changes with the detection of resistance genes (Bhattacharyya et al., 2019).

Colorimetric sensor arrays provide an inexpensive method to detect volatile organic compounds (VOCs) associated with microbial metabolism (Lonsdale et al., 2013; Lim et al., 2014). Specific Technologies Inc (USA) has commercialized the small molecule sensor (SMS) array technology which reacts with the metabolic products of bacteria produced during their growth. Their Reveal-AST printed sensor array system responds to the volatiles emitted during growth producing a colorimetric pattern. It reveals also species ID with 94% accuracy (Sharp, 2020).

The SlipChip/dLAMP technology by Caltech integrates seamlessly cultivation and semiquantitative smartphone-based visual analysis of NA products (Schoepp et al., 2017). This system, currently being commercialized by Talis Inc. (USA), will be described closer in the chapter “POCT-compatible technologies.”

### Whole Genome Sequencing

Progress in Whole Genome Sequencing (WGS) technologies has made them a feasible system for pathogen ID and AST (van Belkum and Rochas, 2018). 3rd generation systems such as Illumina MiniSeq, Pacific Biosciences PacBio Sequel system, or Oxford Nanopore MiniON and PromethION can provide fairly long reads at high speed. In principle, WGS can simultaneously provide fast pathogen ID, epidemiological typing, and detection of drug susceptibility genes. Since WGS provides a massive amount of data in fragmented form, sophisticated software is needed to interpret the results (Quainoo et al., 2017). The European Committee on Antimicrobial Susceptibility Testing reviewed in 2017 the development status of WGS for AST (Ellington et al., 2017). They concluded that, for most bacteria, the available evidence for WGS as an AST tool is still either poor or non-existent and thus inadequate for clinical decision making. They pointed out the urgent need for a single database of all known resistance genes/mutations to facilitate comparison between different systems and bioinformatics tools.

## Proof-of-Concept Technologies

### Smartphone-Based Readers

Kadlec et al. combined smartphone technology to a microphotometric system applying microwell plates coated with antibiotics and the yellow redox indicator dye tetrazolium salt WST-8, which turns orange by the metabolic activity of growing cells. The system could correctly monitor the growth of several pathogens associated with urine tract infection. Samples with concentrations of 10^1^ to 10^6^ cfu/mL could be tested directly without preliminary enrichment cultivations (Kadlec et al., 2014). Feng et al. presented an automated smartphone-based device with a 3D-printed attachment holding a microwell plate. A light-emitting diode array and fiber-based optics enabled detection of turbidity changes in wells already after 1 min (Feng et al., 2016). This system, tested for 17 antibiotics targeting Gram-negative bacteria on clinical isolates of *K. pneumoniae*, provided drug susceptibility interpretation with accuracy of 99.23%. Cui et al. have presented a smartphone-based system for the monitoring of viable bacteria in droplet-based single-cell microdroplet cultures (Cui et al., 2018). In this system, single bacteria were encapsulated in monodisperse microdroplets. This dSPC (Digital Standard Plate Count) platform could quantify *E. coli* and *B. subtilis* in 6 h, compared to 24 h needed for traditional plate counting. These smartphone demonstrations discussed above were performed using pure culture isolates.

Smartphone-based systems applying immunodetection have been demonstrated for ID, but so far not for growth-based AST. Wang et al. introduced a microwell plate-based microphotometric system, which applied a field-of-view adapter and a microprism array between the mobile phone camera and the 96-well plates. In a serological analysis (771 patient samples in 12 serology assays for bacterial/viral infections) the system exhibited 97.59~99.90% analytical accuracy in pathogen identification with costs of ~50 USD per 96-well plate and analytical quality sufficient for POCT (Wang et al., 2018).

An inexpensive (under 100 USD) smartphone-based monitoring system for nucleic acid amplification, smaRT-LAMP, was introduced by Barnes et al. The system contains a hot plate for isothermal amplification, two flexible cables and 96 LED lights fitted into a cardboard box. The Bacticount software detects the emitted green light as a result of a successful amplification and automatically determines the genome copy number in real time. smaRT-LAMP was shown to work well with diverse Gram-negative and Gram-positive pathogens in biological specimens, giving in ~1 h results with matched standard cultivation-based tests. Reliable pathogen ID was obtained for spiked urea and blood samples as well as for urea samples of sepsis patients (10^5^ −10^8^ CFUs). The small sample size (2 μl per reaction), however, limits its use for very diluted samples (Barnes et al., 2018).

Priya et al. have successfully coupled loop-mediated isothermal amplification (RT-LAMP) and sensitive quenching of unincorporated amplification signal reporters (QUASR) detection technologies to visual detection with a smartphone. The portable “LAMP box” was successfully used for the sensitive and specific detection of Zika, chikungunya, and dengue viruses (Priye et al., 2017). This system has not yet been applied to growth-based AST.

### Optical or Microscopic Methods

Choi et al. have developed a rapid antimicrobial susceptibility testing system, dRAST. It can determine the AR from a positive blood culture bottle in 6 h (Choi et al., 2017). The sample is mixed with agarose and inoculated into a well of a plastic microchip. Addition of cultivation medium forms a liquid bridge between the growth chamber and the satellite well, which contains the antibiotic agent. Using microscopic detection of bacterial colony formation in agarose, the total time-to-result was only 6 h with a wide range of bacterial concentrations. The tested clinical isolates (n=206) included 16 Gram-negative species and seven Gram-positive species, and the dRAST system agreed with a standard microdilution test with an accuracy rate of 91.11% (Choi et al., 2017).

Matsumoto et al. described a microfluidic channel method for rapid (3 h) AST for *P. aeruginosa* by automated microscopic detection of cell number and cell morphology (Matsumoto et al., 2016). Their Drug Susceptibility Testing Microfluidic device (DSTM) consisted of five sets of four microfluidic channels and allowed simultaneous microscopic observation. Susceptibilities to the antibiotics (pre-dried into each channel) were evaluated by the differences in cell number and shape between drug-treated and control cells. Hundred and one clinically isolated strains of *P. aeruginosa* tested with DSTM correlated strongly with the results obtained using the conventional microbroth dilution method (Matsumoto et al., 2016). This system waits for applicability testing with other organisms.

In nanowell AST, morphotyping with a phase contrast microscopy and optical signal analysis is performed for 0.5 μl cultures. Antibiotic susceptibility data can be obtained for uropathogens in <4 h. The system showed a total categorical agreement of 97.9% with standard disk diffusion assays, but a careful standardization of cell densities prior to cultivation was found necessary (Veses-Garcia et al., 2018).

### Hybridization Methods

Mezger et al. presented a generic method for rapid species identification and AST after 0.5-2 h cultivation (Mezger et al., 2015). Cultured bacteria from urine samples were lysed by sodium hydroxide and heat, and DNA was captured onto magnetic beads. Padlock probes targeting the 16S rRNA gene were hybridized, ligated and amplified by the circle-to-circle amplification method. Optical imaging system then performed digital quantification. Antibiotic susceptibility profiles of *E. coli* for ciprofloxacin and trimethoprim could be determined with 100% accuracy in 3.5 h (Mezger et al., 2015).

### Detection of Growth-Related Molecules or Antibiotic Degradation Products

Devices detecting volatile compounds, so-called electronic noses (eNose), can recognize a smell-print characteristic for bacterial species and their metabolic profile. Since 1982, electronic noses have been applied in diagnostics (Persaud and Dodd, 1982), but mainly for pathogen ID. The Cyranose system (Smiths Detection) was able to distinguish between controls and samples positive for *S. aureus, S. pneumoniae, Haemophilus influenzae*, and *P. aeruginosa* in upper respiratory tract infections (Lai et al., 2002). Gas chromatography connected to ion mobility spectrometry (GC-IMS E-nose) could reliably distinguish bacterial infections from viral respiratory tract infections (Lewis et al., 2017). Saviauk et al. performed an applicability test for the ChemPro 100i Ion Mobility Spectrometry sensor (Environics Inc.). They could discriminate MRSA from MSSA with 83% sensitivity and 100% specificity (Saviauk et al., 2018) and were able to identify also other pathogens (*P. aeruginosa, Enterococcus, E. coli*, and *Clostridium perfringens*) from culture plates with 78% accuracy. In order to access the AST market, the eNose systems should outperform the simple and inexpensive colorimetric sensor array system “Reveal-AST” (Specific Technologies, USA) which is applicable to growth-based AST.

### NAAT in Growth Monitoring

Real-time qPCR can detect quantitative differences between cultures exposed to various antibiotics and different concentrations. Already a 15 min cultivation can provide a detectable increase of nucleic acids (Schoepp et al., 2017). Although qPCR devices are expensive and require experienced personnel for their operation, low-cost devices based on isothermal amplification might change the game thoroughly. Applying chip electronics and microfluidics, CalTech (the Technical University of California) has developed a device applicable to AST. This system is currently under commercialization by SlipChip Corp and will be described closer later in this review in the section “POC-compatible technology.”

### Biosensor Systems

Biosensors are devices that measure biological or chemical reactions by generating signals proportional to the concentration of an analyte in the reaction. Exposure to antibiotics causes detectable changes in bacterial membranes, morphology, metabolism, movements, mass, heat production and nucleic acid content. In microcalorimetry approaches, heat production correlates with the number of cells arising over time (von Ah et al., 2009). This approach is applicable to both solid and liquid cultures (Howell et al., 2012). Dynamic heat flow patterns have served species identification from urine samples (Bonkat et al., 2012). Isothermal microcalorimetry revealed vancomycin-resistant *Staphylococcus aureus* in <8 h (Entenza et al., 2014). Butini et al. applied isothermal microcalorimetry to real-time monitoring of microbial viability in biofilms in the presence or absence of antimicrobial compounds (Butini et al., 2018). Microcalorimetric methods, although fast and sensitive, require pure cultures and a fairly high number of bacterial cells. In 2017, the Swedish company SymCel announced an extensive 28-months clinical testing of their microcalorimeter calScreener™ for AST. However, currently no clinically validated microcalorimetry systems are available.

For some antibiotics (beta-lactams, chloramphenicol) AMR can be assayed by the follow-up of antibiotic degradation. The BYG Carba test detects conductivity changes caused by the enzymatic hydrolysis reaction of imipenem antibiotics on an electrode coated with polyaniline, which is highly sensitive to changes in pH or redox potential. With a loop-full of bacteria (10 μl) from a fresh plate as a sample, this home-made inexpensive instrument could detect carbapenem resistance in <35 min displaying 95% sensitivity and 100% specificity in comparison to PCR-based analysis (Bogaerts et al., 2016). Mecklenburg et al. developed an assay that directly detects the thermal signal generated from the enzymatic breakdown of antibiotics. The system was able to distinguish between penicillinase and metallo-β-lactamase (Mecklenburg et al., 2017). Its value for clinical work needs to be evaluated, as it requires pure cultures and does not provide pathogen ID.

A variety of electrochemical reporters for cell viability have been applied to viability analysis and drug susceptibility measurements. The system by Besant et al. uses resazurin dye (an oxidation-reduction indicator) to monitor cells trapped in nanoliter wells (Besant et al., 2015). Within 1 h the microfabricated device could detect the response of *E. coli* and *K. pneumoniae* exposed to ampicillin and ciprofloxacin in urine samples spiked with bacteria in concentrations as low as 1 cfu/μL. The level of commercialization of this technology is not known.

In microelectromechanical systems (MEMS) the deflections associated with the micromotions of bacteria attached to a microcantilever provide a signature of bacterial metabolism. Such changes can indicate growth long before the bacteria replicate. With bacteria captured in bi-material microchannel cantilevers, quantitative antibiograms have been obtained for *E. coli* and *S. aureus* within 2 h (Etayash et al., 2016). The bacteria absorb infrared photons and release heat to the support matrix by a process of vibrational energy relaxation, inducing bending of the bimetallic cantilever proportional to the quantity of the released energy. High sensitivity, corresponding to a single cell per μl, was obtained with *Listeria*-containing samples. The researchers plan to further integrate sample separation techniques into this BioMaterial Cantilever platform. LifeScale Analytics (NC, USA) has already launched a commercial product which correlates cantilever vibration to biomass for MIC determination (Burg et al., 2007). This system performs automated cell counting, mass measurement, and visual observation of liquid samples for AST in <3 h, but requires cell concentrations above 10^4^ cells/ml. The company has not yet presented peer-reviewed clinical AST studies for this instrument. Micromotions are affected by flowing liquids, and inefficient transfer of antibiotics to immobilized bacteria can distort the results. Therefore pre-enrichment and pre-purification of bacteria may be necessary (Li et al., 2017b; Syal et al., 2017).

A low-cost and rapid biosensor has been developed employing photoluminescence emission of photo-corroded GaAs/AlGaAs biochips. Growing bacteria protect the biosensor surface against photo-corrosion, while non-growing or dead cells give a higher signal. These biochips exposed to a *E. coli* and *Legionella pneumophila* cultures (w/o antibiotics) were capable of quantifying electrically charged bacteria in 4.5 h (Nazemi et al., 2017).

The Field Effect Enzymatic Detection (FEED) biosensor platform can detect extremely low bacterial concentrations (below 10 c.f.u./ml). An electrical field between the working electrode and the immune complex multiplies the biocatalytic output current, enabling a direct detection of bacteria without sample processing (Shi et al., 2018). These biosensors apply horse radish peroxidase (HRP) as a redox source in the sandwich hybridization complex. Commercial devices applying this technology for AST may potentially emerge in coming years.

### Other Proof-of-Concept Technologies

Flow cytometry (FC) can provide excitation/emission spectra of cells and give information about cell-counts, morphology and viability, enabling AST in 2–3 h. Due to the large amount of raw data, mathematical methods such as adaptive multidimensional statistics must be applied for analysis (Huang et al., 2015). Successful demonstrations with clinical polymicrobial samples are currently missing. Flow cytometric assays struggle with complex patient samples, inefficient staining, the presence of autofluorescence, the inability to differentiate cellular damage for the influence of antibiotics, and lack of clinical databases for validation. Flow cytometry instruments are very costly, making this technology an unlikely candidate for POCT. Atomic force microscope (AFM) allows real time monitoring of bacterial activity. This principle is applicable to both cultivable and non-cultivable cells (Longo et al., 2013), but is an expensive and tedious system for clinical work. Plasmonic imaging and tracking (PIT) can be used for the monitoring of nanometer-scale motions of single bacterial cells before and after antibiotic addition. The observed image contrast fluctuations were found to indicate changes in bacterial metabolism long before cell replication (Syal et al., 2016). Asynchronous magnetic bead rotation (AMBR) is a non-microscopy-based approach capable of monitoring individual cells for elongation, generation time, lag time, division, as well as sensitivity to antibiotics. It has been successfully tested with *E. coli* attached to anti-*E. coli* functionalized beads (Kinnunen et al., 2011). This system is still at the research stage and a complex technology regarding handling, setup and the need for expertise (Schumacher et al., 2018).

## Miniaturized and Chip-Based Growth Monitoring Systems

Lab-on-a-chip systems combine one or several laboratory functions into a single integrated circuit. The microfluidic AST platforms typically apply channels containing pre-loaded dried antibiotics. Following on-chip cultivation, the channels can be monitored with a detection device such as a phase contrast microscope. Some systems use agar to immobilize the bacteria and to form growth chambers. Also gradient-forming microfluidic platforms have been applied (Luka et al., 2015; Campbell et al., 2016). He et al. introduced a chip applying antibody-coated glass beads to capture *E. coli* O157 and to provide fluorescence detection for ID and AST. Their device had an integrated antibiotic release system, which could identify this strain at a range of 10^4^-10^8^ cfu/ml within 30 min (He et al., 2014). Li et al. integrated antibody-coated carbon nanotubes with isothermal amplification. The carbon nanotube multilayer could perform selective capture, cultivation and release of the bacteria. After cultivation the antibody-entrapped bacteria were lysed, and their DNA was accurately quantified by LAMP. This system could detect *E. coli* O157:H7 and its toxin at concentrations as low as 1 cfu/ml without any complicated instrumentation (Li et al., 2017a). Its use for growth-based AST has not yet been presented. Researchers at Hong Kong Baptist University have developed a multidimensional AST system for growth-based AST, providing detection through automated microscopy in 4 h. They established a hydrogel microfluidic chip which simulates drug diffusion and pathogen killing processes inside the human body. Due to the chip's multidimensionality, several antibiotics, nutrients or immunologic substances could be tested simultaneously. This system is currently under commercialization (Sun et al., 2016; Liu et al., 2017a,b). Weibull et al. have presented a nanowell AST device capable of real-time optical reading and growth data analysis. This “stationary nanoliter droplet array” (SNDA) system can provide ID and AST for urine samples within one working shift. A filtering process first isolates bacteria from the clinical urine samples into a growth medium, supplemented with 10% resazurin (oxidation-reduction indicator). Applying 12 bacteria–antibiotic combinations precise MIC determinations could be obtained in 3 h, which is 6-fold faster than traditional broth microdilution assays in 96-well plates. However, follow-up studies with clinical isolates of both Gram-negative and Gram-positive bacteria are needed (Weibull et al., 2014). Veses-Garcia et al. applied 0.5 μl cultures for uropathogens on a 672-nanowell slide equipped with optical signal analysis. They were able to define a precise minimum inhibitory concentration for 70 clinical *E. coli* isolates. Algorithm-assisted optical analysis determined antibiotic susceptibility in 3 h 40 min, showing a total categorical agreement of 97.9% (Veses-Garcia et al., 2018).

The “Integrated Comprehensive Droplet Digital Detection” (IC 3D) system is capable of detecting bacteria directly from diluted blood within 1.5–4 h. It consists of bacteria-specific DNAzyme-based sensors, a droplet microencapsulation system, lysozyme and a 3D particle counter system. The ongoing work aims to develop an automated, portable device for multiplexed and rapid detection of antibiotic-resistant strains (Kang et al., 2014). The Ultrafast Parallelized Microfluidic Platform consists of four arrays (each holding 110 pL droplets with 1–4 bacteria) which are screened by dynamic imaging over 2 h. This imaging-based AST was successfully tested with four types of pathogens causing urinary tract infection (UTI) (Kang et al., 2019).

## POCT-Compatible Devices for id and AST

Systems with a low price and high speed are needed in all healthcare sectors, but especially in outpatient clinics and in developing countries. Point-of-Care Tests (POCT) could bring the diagnostics into outpatient clinics and thus facilitate evidence-based medication in places where extensive prescription of antibiotics happens. Very few rapid AST systems are currently available, but the ongoing development is promising. David Boyle has made an excellent review of affordable technologies and devices applicable to “standard microscopy stations” (Boyle, 2017). The devices presented below can provide a fast pathogen identification. Some of these are already now applicable to AST, and some devices can be even applied to diagnostics of non-infectious diseases as well.

Scanogen Inc. (USA) develops DNA-based (non-amplification-based) “single-molecule biosensors” bound to microparticles, which can convert the hybridization signal to an optical signal. The device contains an inexpensive and low-power light-emitting diode ring and uses disposable sample cartridges, abolishing the need for manual sample preparation. The company is currently developing diagnostic assays for infectious diseases and drug resistance. The MicrobeDx technology (MicrobeDx Inc., CA, USA) applies a transformational ribosomal RNA-based assay on a microfluidic disc platform. The disc holds 150 nucleic acid capture probes spotted onto a glass slide. This system can discriminate four clinically relevant *Staphylococcus* species that differ by a single nucleotide polymorphism (SNP) in diagnostic probe sequences. The protocol includes hybridization, washing, rinsing, and drying steps and does not require purification of the target nucleic acids (Peytavi, 2005). In a clinical testing phase financed by NIH during the years 2018-2019 (so far unpublished), MicrobeDx has tested UroLogic, a highly automated instrument and cartridge system that performs rapid pathogen ID and AST in 30 and 150 min, respectively. The Alveo platform (Alveo Technologies Inc, CA) applies hybridization-based electrochemical nucleic acid detection in single-use cartridges. The cloud-connected device is capable of analyzing 100 infectious diseases. Sample preparation, nucleic acid amplification, and real-time hybridization-based detection are performed in a single isothermal microfluidic channel. This concept has not yet been applied to growth-based AST. The Loopamp™ technology by Eiken Chemical Corp. (China) has already received WHO endorsement for tuberculosis diagnostics (Noncommercial culture drug-susceptibility testing methods for screening patients at risk for multidrug-resistant tuberculosis: policy statement, 2011). The Loop-mediated isothermal amplification (strand displacement reaction) employs four different primers designed for six distinct regions on the target gene. It has been used successfully for the detection of *Neisseria meningitidis* in clinical cerebrospinal fluid samples (Lee et al., 2015) and for the detection of the OXA-23 carbapenemase (Yang et al., 2018) gene of *Acinetobacter baumannii*.

Schoepp et al. (2017) demonstrated with 51 clinical samples that the antibiotic exposure time in phenotypic AST can be shortened to 15 min when dLAMP (digital real-time loop-mediated isothermal amplification) or dPCR (droplet-PCR) is applied. The changes in DNA concentrations (control vs. antibiotic-treated samples) were determined by a “digital single-molecule counting” system after incubation. In dLAMP, the target molecules or lysed cells are partitioned into thousands of nanodroplets so that each compartment contains approximately a single molecule (Schoepp et al., 2017). The 1 nl droplets with DNA concentrations relevant to clinical urine tract infection samples showed 1.23-fold difference in amplification products between resistant and susceptible strains, and 98.1% of the tested samples matched the standard AST results. The LAMP chemistry was optimized and applied to a SlipChip microfluidic device equipped with an electrophoretic system concentrating the bacteria in the sample. This concept was tested for AST of positive blood cultures. The results were consistent with standard microdilution tests or the BD Phoenix System when several broad-spectrum antibiotics and clinical *E. coli* samples as well as the *S. aureus* ATCC 6538 strain were tested (Yi et al., 2019). The Talis Biomedical Corporation (CA, USA) is currently commercializing dAST/SlipChip technology to a product which combines a single-use cartridge integrating fluid partitioning for parallel treatment with different antimicrobials, reagent additions, target lysis, extraction and amplification. The NAAT-based system presented by Priye et al. (2017) applies a reverse-transcription loop-mediated isothermal amplification (RT-LAMP) coupled with the QUASR technique (quenching of unincorporated amplification signal reporters). The device was found to be five times more sensitive than traditional POCT for the detection of the dengue, chikungunya, or Zika viruses. Hassibi et al. have presented a fully integrated, miniaturized semiconductor biochip with closed-tube detection chemistry for multiplex NA amplification and sequence analysis, which they claimed to have a high dynamic quantification range for the microbial load, while at the same time performing comprehensive mutation analysis on up to 1,000 sequences or strands simultaneously in <2 h. This chip was able to correctly detect and quantify multiple DNA and RNA respiratory viruses in clinical samples, while at the same time detecting 54 drug-resistance-associated mutations in six genes of *Mycobacterium tuberculosis* (Hassibi et al., 2018). QuantuMDx Ltd (UK) aims to launch a miniaturized portable diagnostic platform that runs on battery power. This Q-POC™ device would be a portable molecular diagnostic instrument with built-in NA amplification for multiplexed diagnostics and drug susceptibility testing within 20 min. It applies nanowires coated with specific probes for the detection of genetic variants and has cloud-based connectivity to share and utilize epidemiologic data. This technology is currently under clinical evaluation for diagnostics of malaria (protozoan *Plasmodium falciparum*) and tuberculosis (*Mycobacterium tuberculosis*). The device, although not designed for growth-based AST, might have a very wide applicability range including non-infectious diseases such as cancer or genetic disorders. The outcome of the clinical tests has not yet been published. The Multipath instrument (First Light Diagnostics Inc., USA) applies fluorescence-labeled nanoparticles attaching to the target cells by immunobinding. The nanoparticle/cell complex can then be pulled toward the detection system by magnetic beads conjugated with another microbe-specific antibody. Thereby the system is capable of monitoring viable cells using non-magnified digital imaging. A POCT-compatible device is currently under testing. Immunodiagnostic TPX-technology (two-photon excitation fluorometry), commercialized by ArcDia Ltd (Finland) to mariPOC and mariAST platforms, can analyze 40–100 samples a day. It has a cloud-based connectivity allowing efficient sharing of epidemiologic data. TPX allows the use of untreated polymicrobial clinical samples on-line, providing bacterial ID in 20 min and antibiotic resistance in a few hours. Unlike most other AST systems, it can also provide sensitive immunodetection of viruses Sanbonmatsu-Gámez et al., 2015; Bruning et al., 2018.

POCT devices using smartphone technology have long been used for personalized medication applications such as the colorimetric analysis of urine strips (Barnes et al., 2018). The use of smartphones in growth-based AST for miniaturized cultures has been presented in several studies (Kadlec et al., 2014; Schoepp et al., 2017; Cui et al., 2018; Hernández-Neuta et al., 2019), although instrument manufacturers seem to be reluctant to incorporate standard commercial smartphones to their systems. Photolithography, plotting, plasma etching, inkjet printing, cutting, and wax printing can be used to pattern paper for inexpensive diagnostic tests (López-Marzo and Merkoçi, 2016). Paper-based systems have been applied for the immunodetection of *Helicobacter pylori, Chlamydia* infections and Zika virus infections, but platforms integrating LF-testing into growth-based AST have not been presented.

## Problems With Miniaturization and Rapid AST

Due to the small sample size (at lowest a single bacterial cell), rapid AST may not give results representing the whole bacterial population in the diagnostic sample. This problem can be mitigated by analyzing a large number of individual cells. An example of such a strategy is the stochastic confinement of bacteria in nanoliter droplets, so that a high number of individual droplets can be analyzed quickly and efficiently (Boedicker et al., 2008). The direct analysis of clinical samples also demands the use of valid internal standards. Additionally any growth-based analysis may utterly fail if the growth rate is very low or the microbes cease to grow due to the lack of specific growth factors, a non-optimal atmosphere, or the accumulation of growth-inhibiting substances. For intracellular pathogens the use of NAAT may be necessary. Compared to standard microbial cultivations, favorable growth conditions may be difficult to establish for miniaturized set-ups. These problems can be mitigated by analyzing cell viability, morphology or movements. Miniaturization can improve the performance and capacity of cultivation-based AST only if the need for manual steps is minimized. As reagent dilutions are often done before loading onto the chip device, set-up complexity may stay a similar level as in broth dilution methods. Due to the relatively low number of pathogens in most clinical samples and the impurities of the sample matrices, miniaturized cultivations benefit from methods concentrating the cells and removing the impurities. The FISH-based Accelerate Pheno system applies electrokinetic/electrophoretic system for these purposes. Coarse cell sorting for blood samples can be achieved through simple inertial microfluidic systems, where centrifugal (inertial) force drives bacteria to the outer side of a spiral-formed microcapillary tube, while blood cells stay on the inner side (Bhattacharyya et al., 2017). Some systems bypass the problem of impurities by bringing the target close to the biosensor surface with magnetic and antibody-coupled beads or nanoparticles. Miniaturization sets high demands to the standardization of the conditions, as the samples should represent similar growth states and culture densities. Robots may be needed for accurate pipetting.

Serious doubts concerning the ability of accelerated cultures to fully substitute standard growth-based tests have been presented. Accelerated cultivations may struggle to differentiate between wild type and resistant strains (Leclercq et al., 2013; Kahlmeter, 2014; Maurer et al., 2017). This is especially a concern in the case of induced expression of the resistance factors, e.g., in the detection of AmpC in enterobacteria or macrolide-resistance in streptococci (Leclercq and Courvalin, 2002; Jacoby, 2009; Harris and Ferguson, 2012).

## Economic Drivers for Rapid AST

Substantial overall savings can be obtained with rapid AST through the reduction of hospital days, disability days and the saving of lives (Cassini et al., 2019). Despite the potential total cost savings, the high price of molecular testing forms an efficient barrier for the installation of new technologies. A price tag ranging from $100 to $250 per test is common for molecular tests (Li et al., 2017b). For a typical 500-bed community hospital, detailed multiplex testing of positive blood cultures by NAAT could cost more than $500,000/year in reagents alone (She and Bender, 2019). Also the instrument prices may be significant. The total costs of testing are quite difficult to estimate. The prices of test kits and diagnostic instruments are based on the distributors' offers. Testing costs include clinical sampling and usually also cultivations in microbiology labs (enrichment, preparation of bacterial isolates) before AST can be performed. As an example, Patel et al. estimated the total costs for mass spectrometry-based AST to be roughly 79 € per patient, when the cost of the MALDI-TOF device, reagents, pharmacist time and the antimicrobial stewardship program are pooled together (Patel et al., 2017). Yet the reagent costs for one MS-sample can be pressed close to 1 €. A typical MALDI-TOF MS system with all the accessories, software databases and maintenance is costly (up to €200,000 per year) (Wieser et al., 2012). This implies that the use of the instrument must be high for an acceptable cost efficiency. This unfortunately rules out their use in outpatient clinics. MS-instruments are, however, very versatile and can be applied also to other routine diagnostics in central laboratories (Vrioni et al., 2018). Direct inoculation from plates to automated identification systems such as Vitek, Microscan and others has been validated and used in many clinical laboratories. These systems apply multiwell liquid cultures and have a turnaround time as short as 4 h for ID and 6–8 h for AST (She and Bender, 2019), but the prices of these instruments are high. The cost of any rapid AST should not significantly exceed the cost level currently considered acceptable for routine testing: this might be roughly 30 to 50 €, including sampling, culturing and AST (EU, 2013). Five phenotypic tests for a targeted detection of enterobacterial carbapenemases have been recently evaluated for their performance and costs. All these tests required the use of culture isolates. Per sample, the costs of multiplexed PCR-based analysis was 30 €, immunochromatographic methods ~15 €, a colorimetric assay 5 € and carbapenem hydrolysis test 1 € per sample (Baeza et al., 2019). Apart from turnover time, also the capacity of the test system is important. Among automated microscopy systems, Accelerate Pheno handles one sample per unit module (max. four modules per device, cost per sample ~250 €), whilst the Q-Linea ASTar system can handle 50 samples per day. The capacities of NAAT-based systems can be even higher. Automated MIC-determination systems such as VITEK-2 can manage tens of samples per time. For POCT usage, however, a throughput capacity of a few tens of samples per time would be sufficient.

## Summary: How do the AST Technologies Meet the Requirements?

Several identification systems based on NAAT, NA hybridization and immunodiagnostics are already available for rapid diagnostics, but only few are applicable to POCT. This is due to high costs, lack of appropriate facilities or expert labor, or insufficient performance with clinical samples. Multiplexing and high-throughput capacities are important for central laboratories, but most healthcare settings process only few samples per time. POCT use rules out expensive devices, systems requiring demanding sample preparation, and systems requiring standard microbiology laboratory facilities. NAAT serves well for the identification of viral and bacterial pathogens, and in some cases provides also the detection of AR genes. However, the commercial multiplexed diagnostic NAAT panels are far too expensive for routine diagnostics. Immunochromatographic tests, especially dip-sticks, are a low-cost and handy option for virus diagnostics and detection of inflammatory factors. Unfortunately systems capable of detection of antibiotic resistance proteins directly from clinical samples without enrichment cultures, culture isolation or sample purification do not yet exist. The routine diagnostic tools for infectious diseases should serve a wide field of requirements: both the identification of the pathogen (viral and bacterial), the detection of antibiotic resistance and the determination of the correct antibiotic dosing. Systems based on microscopy are not compatible with the detection of viruses or inflammatory factors. Diagnostic systems differ very much in their potential to provide all relevant data for medication (Table 1). Multiplexing NAAT-based systems can seamlessly incorporate the detection of emerging resistance-related genes or mutations. However, every update for a diagnostic panel requires new validations. While central laboratories have resources to validate new tests against the standardized tests, smaller healthcare units are bound to use tests validated by authorities such as the FDA. The few systems currently capable of providing simultaneous ID and growth-based AST directly from clinical samples are based either on FISH (Accelerate Pheno), immunodiagnostics (e.g., mariAST, MultiPath, immunobiosensors), or digital AST (dAST) (Table 1). As their properties and application areas differ, all these systems may have a good future. Rapid growth-based diagnostic systems rely on accelerated cultivations. For this reason, it is necessary to validate every new technology carefully against the standard tests accepted by EUCAST and CLSI. The MIC breakpoints (sensitive, resistant, or in-between) must be re-defined for each system and each tested organism. This requirement definitely retards the deployment of new technologies. While most novel AST systems only perform an endpoint analysis, the systems based on microscopy, heat production, movements, immunodetection or detection of mass changes in principle facilitate on-line monitoring. Biosensor-based AST systems, however, yet miss convincing clinical demonstrations.

**Table 1:** Properties of technologies applicable to rapid identification and AST.

**Technology**	**Company or product (examples)**	**Time for AST (h)**	**Simultaneous ID and AST**	**Clinical polymicrob. samples**	**Online AST**	**Provides MICs**	**Detects new resistances**	**AST for non-culturable microbes**	**Enables virus ID**	**Level of commercialization**	**References**
**Standard cultivation tests**											
Broth dilution test	Several	18–36	–	–	✓	✓	✓	–	–	Gold standard	
Disk diffusion test and E-test	Several	18–24	–	–	✓	✓	✓	–	–	Gold standard	
**Automated readers for cards or microtiter plates**											
Broth microdilution-based instruments	bioMerieux, BD, Siemens	5–16 h	–	–	✓	✓	✓	–	–	Commercial	
Disk diffusion-based instruments	Giles Scientific, Oriana, BioRad, BD	5–16 h	–	–	✓	✓	✓	–	–	Commercial	
**Mass spectrometry (biochemical profiling, follow-up of antibiotic degradation, detection of anti-microbial protein)**											
MALDI-TOF (Bruker MBT)	Bruker, Shimadzu, Sciex, Waters	2-4 h	–	–	–	–	✓	–	–	Commercial	
MBT-ASTRA (biochem. profiling after antibiotic exposure)	Bruker Daltonik GmbH	2-4 h	–	–	–	✓	✓	–	–	Commercial	Sparbier et al., 2016
Direct-On-Target Microbial Growth Assay (DOT-MGA)	All instrument providers	4 h	–	–	–	✓	✓	–	–	Experimental	Idelevich et al., 2018; Correa-Martínez et al., 2019
**Fluorescence & hybridization**											
FISH (fluorescent probes, microscope)	XpressFISH	2–4 h	✓	✓	–	–	✓	–	–	Commercial	Salimnia et al., 2014
Multiplexed automated microscopy/FISH	Accelerate Diagnostics	6.5 h	✓	✓	✓(fs)	✓	✓	–	–	Commercial	Hill et al., 2017
Automated fluorescence detection for expression profiling	NanoString Technologies	24 h	✓	✓	✓(fs)	✓	✓	–	✓	Commercial, under testing	Barczak et al., 2012; Bhattacharyya et al., 2017, 2019; Kelley, 2017; Koehler et al., 2018
Non-microscopic imaging, fluorescent antibody-bound nanoparticles, magnetic beads for concentrating	First Light Diagnostics	4 h	✓	✓	✓	✓	✓	–	✓	Commercial, under testing	https://www.firstlightdx.com/publications/
**Other imaging or spectroscopy-based systems**											
Surface plasmon resonance (SPR)	Biacore 3000 and exp. devices	0.5–4h	–	–	✓	✓	✓	✓	–	Experimental	Chen et al., 2011; Tao and Syal, 2016
Raman spectroscopy (SERS)	Several	2h	✓	–	✓	✓	✓	–	–	Experimental	Liu et al., 2016; Wang et al., 2018
Smartphone-based growth monitoring of microplates, capillaries or chips	Experimental	2–4 h	–	–	✓	✓	✓	–	–	Experimental	Kadlec et al., 2014; Feng et al., 2016; Cui et al., 2018
**Cell sorting systems, flow cytometry**											
Flow cytometry	FASTinov	2 h	–	✓	✓	✓	✓	(✓)	–	Commercial	Costa-de-Oliveira et al., 2017
**Sensor-based detection of micromotions or mass changes**											
Plasmonic imaging and tracking for nanomotions	Experimental	<1 h	–	–	✓	✓	✓	✓	–	Experimental	Syal et al., 2016
Atomic force microscopy cantilever	Experimental	0.25-4h	–	–	✓	(✓)	✓	(✓)	–	Inverted microscope	Longo et al., 2013
SAW and other mass sensitive biosensors	Experimental	0.5-6h	–	–	✓	(✓)	✓	(✓)	✓	Experimental	Chang et al., 2007; Hoß and Bendas, 2017
**Heat production**											
Microcalorimetry	SymCel AB, TA Instruments	Few hours	–	–	✓	(✓)	✓	(✓)	–	Commercial	https://www.laboratoryequipment.com/article/2017/11/how-use-calorimetry-tackle-antibiotic-resistance
**Immunochromatography (lateral flow tests, “dip-sticks”)**											
Resistance factor specific binders	Coris Bioconcept	0.25–4	–	–	–	–	–	–	–	Commercial	ECCMID 2015 Booth #243
**Immunodetection, fluorescence-based**											
Two-photon fluorescence microscopy TPX	ArcDia	2–4 h	✓	✓	✓	✓	✓	–	✓	Commercial, under testing	Koskinen, 2008
Multipath (magnetic beads, non-microscopy imaging)	First Light Diagnostics	2–4 h	✓	✓	✓	✓	✓	–	✓	Commercial, under testing	
**Electrochemical biosensors or detection of volatile organic compounds**											
rRNA-hybridization, peroxidase signaling	GeneFluidics	2–5 h	✓	✓	–	✓	✓	(✓)	–	Commercial	Mach et al., 2011; Liu et al., 2014
Colorimetric sensor array for VOC detection	Specific Diagnostics	3–4 h	✓	–	✓	✓	✓	–	–	Commercial	https://www.specific-dx.com/reveal-ast
Redox-indicator resazurin	Experimental	1 h	–	–	✓	✓	✓	(✓)	–	Experimental	Besant et al., 2015; Avesar et al., 2017
Field effect enzymatic immunosensor	Experimental	1–2 h	✓	✓	✓	✓	✓	(✓)	–	Experimental	Shi et al., 2018
Electronic nose: ion mobility spectrometry sensor	Environics, Olfactomics	Few minutes	(✓)	–	(✓)	–	–	–	–	Commercial	Lewis et al., 2017; Saviauk et al., 2018
**NAAT**											
PCR, qPCR	Several	2–4 h	✓	✓	–	–	–	✓	✓	Commercial	
Integrated cassette-based NAAT solutions	Several	4 h	✓	✓	–	–	–	✓	✓	Commercial	
Isothermal amplification	Several	0.5–4	✓	✓	–	–	–	✓	✓	Commercial	
Whole Genome Sequencing	Several	1–24 h	✓	✓	–	–	✓	✓	✓	Commercial	
**NAAT combined to cultivation**											
Isothermal amplification, digital AST	Talis Biomedical	0.5 h	✓	✓	✓(fs)	✓	✓	(✓)	✓	Under commercialization	Schoepp et al., 2017, 2020; https://talis.bio

*The marking ✓ is in brackets, if the possible feature lacks experimental demonstrations. – indicates a missing property. fr indicates the need for frequent sampling*.

In practice clinical diagnosis should be obtained during office hours, meaning <8 h from sampling to results. This does not leave time for enrichment cultivations or preparation of culture isolates. The strategy “take a sample, store and analyze later” works fine for diagnosis of slowly advancing infections, but also for NAAT-based systems which do not require viable microbes. Growth-based AST, on the other hand, need fresh samples to ensure pathogen survival until the point of analysis.

Regarding the speed and the need to handle clinical polybacterial samples, the immunodiagnostic TPX-technology (ArcDia Ltd), growth-based FISH (Accelerate Pheno), the Multipath digital imaging technology based on nanoparticles for labeling and magnetic beads for capturing (First Light Diagnostics Inc.), and the NAAT-based dAST (Talis Inc.) seem promising options for rapid point-of-care testing of antimicrobial susceptibility. The Talis system is, however, not yet on the market. Immunobiosensors are still in their infancy, but may in the future become important in testing non-culturable microbes. Lateral Flow (immuno-chromatographic “dip-sticks”) systems would be an ideal product format for clinical work, but their applicability has been so far proven only with isolated cultures (i.e., colonies on plate).

Due to the plethora of different resistance mechanisms, NAAT struggles with the detection of antibiotic resistances in Gram-negative bacteria (Maurer et al., 2017) and with the analysis of samples containing commensal flora. NAAT may fail to identify several ESBL genes and genes providing fluoroquinolone or aminoglycoside resistance. NAAT is, however, a powerful and necessary technology for the detection of fastidious, slow-growing or intracellular pathogens, toxin-producing bacteria such as *E. coli* O157:H7, and viruses (Miller et al., 2018). Still, the increased use of NAAT has raised a concern about the fate of bacterial samples required for further studies (Marder et al., 2017; McAdam, 2017). Since NAAT does not require viable samples, subsequent epidemiologic studies or cultivation-based confirmatory AST may be impossible with the stored samples. Whole genome sequencing (WGS) is still in its infancy regarding its use for rapid AST. The required bioinformatics is challenging, and universal open databases are needed to interpret the results.

In the near future, the progress in chip, microfluidics and biosensor technologies may provide new inexpensive AST systems. Integration of many sophisticated technologies will be needed to resolve problems with a low initial pathogen number and the presence of contaminating sample matrices. Several scientific publications have already demonstrated the successful use of smartphone optics and telecommunication capacity for monitoring of microwell or microcapillary cultivations, pH and redox changes and for delivering the read-outs of biosensor data (Berg et al., 2015; Feng et al., 2016; Cui et al., 2018; Hernández-Neuta et al., 2019). The obvious lack of IPR protection for smartphone-based analytic devices and the requirement to validate analytic devices as an entity unfortunately discourages commercialization of these technologies.

## Conclusions

Standard growth-based technologies based on disc diffusion and broth dilution still dominate in AST. They are slow and require pure cultures, but in other aspects serve the purpose well. Only few rapid growth-based AST methods work directly with polymicrobial clinical samples, which is required in POCT. Sensitive growth monitoring can be achieved either by frequent sampling (applicable to disruptive methods like FISH or NAAT) or by on-line immunodiagnostic methods. dAST with chip-based microfluidics devices and isothermal amplification can potentially revolutionize phenotypic AST. With this approach, thousands of individual single bacterium droplet samples can be categorized according whether the amount of amplified DNA reaches the limit defined for growing cells. The FISH-based Accelerate Pheno system has already reached FDA approval. The mariPOC device based on immunodetection with two-photon excitation fluoroscopy allows non-disruptive microbial identification. Its clinical validation for AST should be followed with interest, as this technology enables the use of non-purified clinical samples and also allows follow-up studies to confirm the results. Additionally it already provides a rapid and sensitive identification of both bacterial and viral pathogens. The Multipath technology (First Light Diagnostics Inc.) may provide a functional platform for automated on-line detection by applying fluorescent nanoparticles for signaling, magnetic beads for binding, and non-microscopic imaging for detection. Multiplexing cartridge-based NAAT solutions are likely to reach a significant customer base in central laboratories, since they offer high speed, work well with non-culturable bacteria and viruses and possess an excellent high-throughput power for pathogen ID. However, all new emerging technologies struggle to meet all of the criteria Prof. Kahlmeter set for AST technologies (Table 2). As none of the presented technologies is optimal in all aspects, it is probable that many of them will reach a large customer base. Therefore the consensus statement of the PIAMR AMR- RDT Working Group on Antimicrobial Resistance and Rapid Diagnostic Testing is still valid: “*There is no single major, or broadly accepted, technological breakthrough that leads the field of rapid AST platform development”* (van Belkum et al., 2019b).

**Table 2:** Contemplation on Prof. Kahlmeter's criteria for new technologies (Kahlmeter, 2016).

**Criteria**	**Contemplation**
Generally applicable or restricted to certain infections?	In principle all growth-based rapid AST systems are generic and work with culture isolates. However, for polymicrobial clinical samples they must be coupled with specific probes or antibodies which provide ID. Therefore, specific test panels have been developed e.g., for respiratory, urinary, and blood samples. The pathogen load may not be high enough for direct analysis, and especially blood samples may require culturing prior to analysis. AST for fastidious, non-culturable, or intracellular pathogens call for NAAT. The complexity of the sample matrix affects the choice of the diagnostic system and the methods for sample preparations.
Capacity: how many organisms/agents per hour can be processed	DNA-arrays and PCR systems (including multiplexed cassette designs) have a high throughput capacity. Mass-spectrometry performed on PCR products can handle hundreds of samples per hour in central laboratories. In outpatient clinics speed is more essential than the capacity. High multiplexing (parameters per sample) and high-throughput capacity (number of samples) may be challenging to combine. Progress in NAAT, immunodiagnostics, biosensor technologies and microfluidics has yielded several systems capable of analyzing tens of samples per day or even during a single work shift.
Has the technology been validated against reference methods?	So far quite few quick technologies have received FDA-approval. Currently they include PCR-tests, cartridge-based NAAT-systems and Accelerate Pheno (automated microscopy). Several clinical trials are in progress to achieve CE-marking or FDA-clearance.
Are there any reference installations?	Commercial analysis systems in general do have, and manufacturers tend to publish successful clinical trials. However, finding a lab which lines up to the specific needs may be challenging.
Is scientific literature available?	For mature commercial systems scientific references can be fairly easily found. For near-market products this is much more challenging. Companies often only declare on-going tests, but provide only limited info about the progress. Scientific articles typically present proof-of-concept level data obtained with isolated cultures spiked into sample matrices.
When on market?	Many systems are already available, but they may have a limited scope for ID/AST. Due to lack of clinical data, some systems have a “research use only” status. Some have been accepted only for veterinary use. Commercially mature products include several NAAT systems, FISH-systems and immunodiagnostic system.

## Author's Note

The described technologies have been taken into consideration without any pre-selection and have been judged only by their applicability to clinical diagnostics.

## Author Contributions

AV was the main responsible for the acquisition and structuring of the scientific literature. The text and conclusions were processed by all the authors. VH and OL mediated valuable contacts to the expert biotech scientists and healthcare experts consulted for this review. All authors contributed to the writing and accepted the final version.

## Conflict of Interest

VH was employed by Fimlab Laboratories. The remaining authors declare that the research was conducted in the absence of any commercial or financial relationships that could be construed as a potential conflict of interest.

## Abbreviations

AMR: Antimicrobial Resistance
AR gene: Antibiotic Resistance gene
AST: Antimicrobial susceptibility testing
CLSI: Clinical &
Laboratory Standards Institute
EUCAST: European Committee on Antimicrobial Susceptibility Testing
CFU: Colony forming units
FISH: Fluorescence *in situ* Hybridization
ID: Identification
LF: Lateral flow
LCR: Ligase Chain Reaction
MALDI-TOF: Matrix-Assisted Laser Desorption/Ionization Time-Of-Flight mass spectrometry
MIC: Minimal Inhibitory Concentration
POCT: Point of Care Testing
NA= Nucleic Acid
NAAT: Nucleic Acid Amplification Technology
PIT: Plasmonic Imaging and Tracking
SERS: Surface Enhanced Raman Scattering
FEED: field effect enzymatic detection
MADM: Multiplexed Automated Digital Microscopy
PNA: Peptide-Nucleic Acid probe
WGS: Whole Genome Sequencing.
